# Optic disc melanocytoma with normal tension glaucoma and angle closure glaucoma: Two case reports

**DOI:** 10.1097/MD.0000000000021350

**Published:** 2020-07-24

**Authors:** Dae Sung Kim, Hae Min Park, Han Woong Lim, Won June Lee

**Affiliations:** a Department of Ophthalmology, Hanyang University College of Medicine, Seoul, Korea; b Department of Ophthalmology, Hanyang University Guri Hospital, Seoul Korea; c Department of Ophthalmology, Hanyang University Seoul Hospital, Seoul, Korea.

**Keywords:** glaucoma, melanocytoma, optic disc

## Abstract

**Rationale:**

Optic disc melanocytoma is an ophthalmic tumor that arises from melanocytes, and is a variant of the melanocytic nevus. Here we report 2 cases of optic disc melanocytoma in Asian patient: one associated with normal tension glaucoma (NTG), and the other associated with angle closure glaucoma (ACG).

**Patient concerns:**

Case 1 is a 57-year-old Asian female presented to our department for a general ophthalmic examination. Incidentally, brownish pigmented lesion was found on dilated fundus examination of her right eye. The fundus examination and optical coherence tomography (OCT) examination revealed a mass within optic disc, and superotemporal retinal nerve fiber layer (RNFL) thinning. The Humphrey visual field test showed corresponding visual field defect. Fluorescein angiography showed no leakage around the lesion. Case 2 is a 78-year-old Asian woman presented with complaints of acute bilateral ocular pain. The initial examination revealed shallow anterior chamber. Under the impression of intermittent angle closure attack, prophylactic laser peripheral iridotomy were performed. On dilated fundus examination, black pigmented lesion was found at superior sector of optic disc. Further examination revealed bilateral superotemporal, inferotemporal RNFL thinning on OCT, and spatially corresponding visual field defects.

**Diagnoses:**

Clinical diagnosis of NTG was made for case 1 patient. Although it was a little distant from typical glaucomatous changes, nevertheless she had RNFL defect compatible with visual field defects. Considering her normal IOP and angle structures, we believe NTG was a probable diagnosis for the patient. In case 2, we made diagnosis of ACG presenting as intermittent angle closure attack because of her presenting symptoms, narrowing of anterior chamber and angle structures found on gonioscopic and slit lamp examinations.

**Interventions:**

In Case 1, we prescribe 0.005% latanoprost ophthalmic solution. In Case 2, at first prophylactic laser peripheral iridotomy was performed. Then, topical eyedrops administration was started, and the patient was examined periodically.

**Outcomes:**

In Case 1, at 6 months’ follow-up, OCT and visual field test showed no progression. In Case 2, to this date, the optic disc melanocytoma remains stable for over a 6-year-follow-up period.

**Lessons:**

The fact that NTG and ACG can coexist in patients with melanocytoma of optic disc should be recognized, and the possibility of such should appropriately be evaluated.

## 1 Introduction

Melanocytoma is a benign melanocytic lesion that arises from uvueal melanocytes of neural crest, and is considered congenital.^[1,2]^ The clinical entity was regarded as malignant until it was later revealed to be benign at 1962 by Zimmerman. Melanocytoma typically occurs in the area of optic disc, and it rarely occurs in ciliary body, iris, choroid, even conjunctiva.^[3,4]^

Although there has been several case reports regarding melanocytoma found within optic disc, there were only a few reports on its association with glaucoma.^[5–7]^ Hence, we intend to report 2 cases of optic disc melanocytoma that were associated with glaucoma in Asian patients: one associated with normal tension glaucoma (NTG), and another associated with angle closure glaucoma (ACG).

## 2 Case report

### 2.1 Case 1

**Figure 1:**
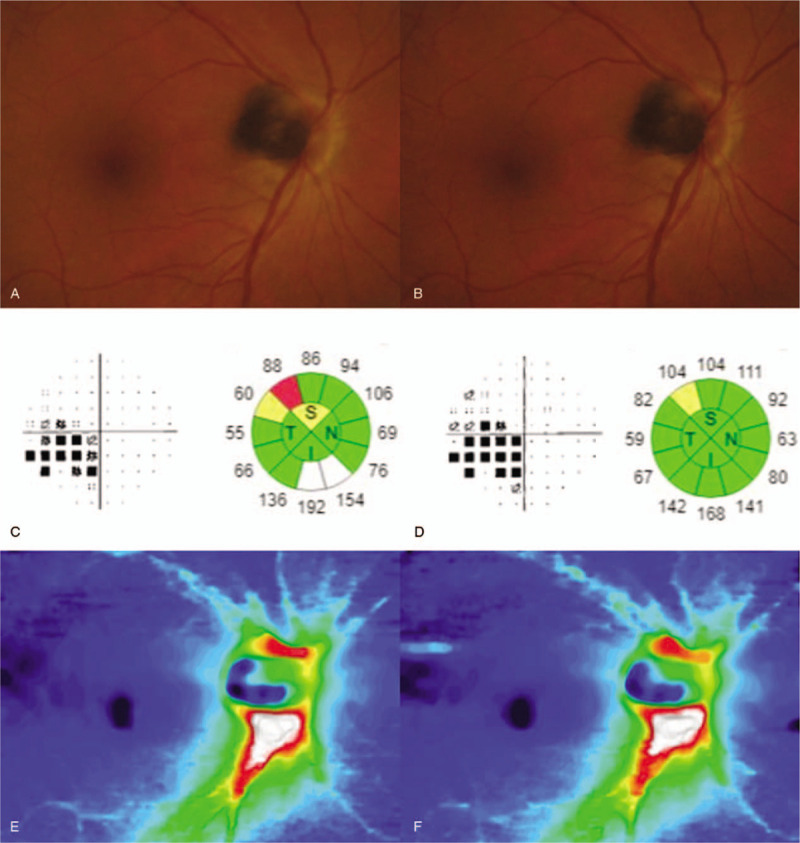
Fundus photographs and the Humphrey visual field test (30–2, pattern standard deviation) and optical coherence tomography (OCT) results of patient at initial visit and 6 month follow up. (A) In case 1, brown peripapillary lesion was found at initial fundoscopic examination. (B) No changes in size were observed over 6-month follow-up period. (C) At initial visit, OCT showed superotemporal retinal nerve fiber layer (RNFL) thinning, and visual field examination showed inferior nasal step corresponding to it. (D) Progression of the visual defect and RNFL thinning was not observed over 6-month follow-up period. (E and F) RNFL thickness map at initial visit and 6-month follow-up visit showed no change in RNFL thickness.

A 57-year-old Asian female presented to our department for a general ophthalmic examination. Her medical history and review of systems were unremarkable. On initial examination, her best corrected visual acuity was 20/20 OD in both eyes, intraocular pressure (IOP) was 14 mmHg in the right eye and 18 mmHg in the left eye. Other than mild cataract, slit lamp examination was within normal limits. Furthermore, there was no peripheral anterior synechiae and widely opened angle was observed on gonioscopic examination. On dilated fundus examination, brownish pigmented lesion on optic disc was found. The optical coherence tomography (OCT) examination revealed a mass within optic disc, and superotemporal retinal nerve fiber layer (RNFL) thinning. The Humphrey visual field test showed an inferior nasal step corresponding to superotemporal RNFL defect. Fluorescein angiography showed neither any filling delay in early phase, nor any peripapillary leakage in early and late phase. Thus, under the diagnosis of NTG in right eye, we prescribe 0.005% Latanoprost (Xalatan) ophthalmic solution. At review at 6 months, melanocytoma remained stable, with no evidence of tumor growth. Further superotemporal RNFL thinning, deterioration of visual function was not observed in follow-up OCT, visual field test (Fig. 1).

### 2.2 Case 2

A 78-year-old Asian woman presented with complaints of acute bilateral ocular pain. At examination, her uncorrected vision was 20/25, and her best corrected vision was 20/20 in both eyes. IOP as measured by Goldmann applanataion tonometry, was 19mmHg in the right eye and 19 mmHg in the left eye. On slit lamp examination, both eyes were normal except a very shallow anterior chamber (Shaffer grade 2 by gonioscopy) of her right eye. Axial length was somewhat short as it was measured to be 22.84 mm in the right eye and 22.89 mm in the left eye. Under the impression of intermittent angle closure attack, immediate prophylactic laser peripheral iridotomy (LPI) was performed.

**Figure 2:**
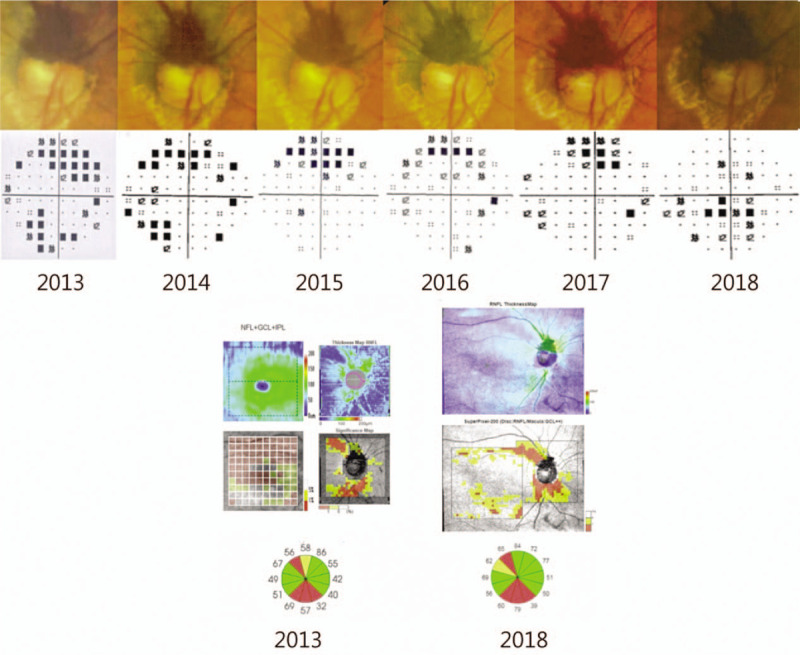
Above image shows fundoscopy image that were performed yearly for 6-year follow-up period, and Humphrey visual field test (30–2 pattern standard deviation). Yearly fundoscopic examinations showed no significant change in size of papillary melanocytoma. Visual field test showed no progression of defects. The bottom image shows RNFL thickness map and peripapillary RNFL and macular GCIPL significance map of patient at initial visit and 6-year follow-up. At superotemporal and inferotemporal RNFL thinning showed at initial visit. There is no significant change in RNFL thickness at 6-year follow-up.

After a successfully performed LPI, dilated fundus examination was performed, in which black pigmented lesion was found at superior sector of optic disc. Further examination revealed bilateral superotemporal, inferotemporal RNFL thinning on OCT, and spatially corresponding visual field defects. Dorzolamide-Timolol (Cosopt) and Brimonidine (Alphagan) administration was since started, and the patient is examined periodically. To this date, the optic disc melanocytoma and RNFL thickness remain stable for over a 6-year-follow-up period (Fig. 2).

## 3 Discussion

Originally, melanocytoma was thought to be malignant due to its morphological similarity to melanocytosis.^[1]^ In 1965, Zimmermann reported 2 white cases of iris melanoma, in which it was later revealed to be iris melanocytoma after enucleation. Melanocytoma, in many cases, is asymptomatic and found coincidentally. However, impairment of visual acuity can occur in 26% of the cases due to retinal exudate involving fovea or neuroretinitis, secondary to tumor necrosis. In rare instance, severe visual impairment can occur due to spontaneous ischemic necrosis, malignant change or secondary retinal vein occlusion. Enucleation is usually considered in such cases, but it should be recognized in advance that the impairment can be induced by transient ischemic necrosis or tumor-promoting inflammation, and that it can later show improvement.^[1,8]^ Intraocular melanocytoma typically occurs within optic disc, but there were only a few reports on its association with glaucoma. Hence, we intend to report 2 cases of optic disc melanocytoma associated with NTG and ACG, respectively.

Visual field abnormalities are seen in upto 90% of optic disc melanocytoma patients, commonly without any significant decline in visual acuity. It can manifest as an enlarged blind spot when the tumor extends beyond the disc margin, whereas arcuate defect is related to compression of nerve fiber bundle. This visual field impairment is known to change with increase in tumor size, but there has been no reports that studies changes of visual field defect according to increased tumor size in Asian population.^[1]^ Similarly, no significant change in tumor size and corresponding visual field alterations was observed in our cases as well.

In western population, in a review of 116 cases, Shield et al reported that minor tumor enlargement was observed in 11% of the patients within 5 years, and 32% within 10 years.^[6]^ According to a study of optic disc melanocytoma in Korean population, it was observed that minor tumor growth was observed in 0% of the patients by 1 year, 5% by 2 years, 14% by 5 years and 57% within 8 years. An average tumor enlargement of 1.8 mm was observed in 53 months of observation.^[9]^

After a thorough literature review, we recognized that our first case is the first reported case of NTG associated with optic disc melanocytoma in Asian patient, other than a case of NTG associated with optic disc melanocytoma recently reported by a Japanese research group.^[10]^ However, there a few limitations to case 1. First, since optic disc melanocytoma is able to induce compressive RNFL defects and subsequent visual field defects that is similar to NTG, the possibility of such cannot be completely excluded in the first case. Second, glaucoma, by definition, is a progressive optic neuropathy characterized by corresponding loss of vision. This entails a long term follow up study is required to make a definite diagnosis of NTG in this patient. In the second case, ACG was associated with optic disc melanocytoma, and to our knowledge, this is the first report of such instance. A case of secondary acute angle closure attack has been reported that was caused by neovascular glaucoma due to retinal vein occlusion secondary to tumor necrosis. However, the relevance between optic disc melanocytoma and ACG has not yet been established.^[11]^

In the second case, the patient had shallow anterior chamber depth and short axial length. It suggests a possibility of coincidental occurrence of 2 clinical entities, due to high ethnical prevalence of glaucoma in Asians.^[12]^ However, ACG in this case may also have been caused by microscopic invasion of melanocytoma into iris or ciliary body, which may not be evident on gonioscopic examination. Therefore, different imaging modalities, such as ultrasound biomicroscopy, may be required to meticulously inspect anterior chamber and structures behind it.

As it was mentioned above, RNFL and visual field defects can be caused by compressive effects of optic disc melanocytoma. Hence it is necessary to differentiate whether the RNFL defect and corresponding visual field changes are due to glaucoma itself or compressive effects of optic disc melanocytoma, though it is very challenging to do so. It would also be important to distinguish whether the visual field progression during follow-up period is due to tumor growth or glaucoma itself. In recent studies, OCT angiography has been used to study decreased perfusion around RNLF layer.^[10,13]^ Recently, one group reported the case series showing that circulatory disorder caused by the optic dis melanocytoma might be correlated with visual field defect.^[14]^ Therefore, in optic disc melanocytoma patients, long-term monitoring of visual field changes and RNFL, perifovea, ganglion plexiform layer with OCT angiography is necessary. In case of disease progression, correlation between changes of tumor size and visual field defects should be analyzed, to detect progression of either one or both of the conditions.

## 4 Conclusions

To conclude, 2 cases of optic disc melanocytoma associated with glaucoma, one with NTG and the other with ACG, were presented in this report. A physician should always keep in mind the possibility of the 2 disease entities occurring concurrently, glaucoma evaluation should be performed for such patients for initial and follow-up visits.

## Author contributions

**Conceptualization:** DSK, HMP, HWL and WJL

**Data curation:** DSK, HMP, HWL and WJL

**Formal analysis:** DSK, HMP, HWL and WJL

**Funding acquisition:** HWL and WJL

**Investigation:** WJL

**Methodology:** WJL

**Project administration:** WJL

**Resources:** WJL

**Supervision:** WJL

**Validation:** WJL

**Visualization:** WJL

**Writing – original draft:** DSK, HMP, and WJL

**Writing – review & editing:** HWL and WJL
